# Supplementary material for: Bioorthogonal Metabolic Labeling of the Virulence Factor Phenolic Glycolipid in Mycobacteria
Source: ACS Chem Biol. 2024 Mar 5;19(3):707–17. doi: 10.1021/acschembio.3c00724 (PMC10949201; doi:10.1021/acschembio.3c00724)
Supplement: Supplementary file 1 — cb3c00724_si_001.pdf [file cb3c00724_si_001.pdf]

Supporting information for

Bioorthogonal metabolic labeling of the virulence factor phenolic glycolipid in mycobacteria

Lindsay E. Guzmán[‡], C. J. Cambier[‡], Tan-Yun Cheng[†], Kubra F. Naqvi[⊥], Michael U. Shiloh[⊥], D. Branch Moody[†], Carolyn R. Bertozzi[‡*]

‡Stanford Sarafan ChEM-H, Stanford University, Stanford, California 94305, United States; Department of Chemistry, Stanford University, Stanford, California 94305, United States.

†Brigham and Women's Hospital, Division of Rheumatology, Inflammation and Immunity, Harvard Medical School, Boston, Massachusetts 02115, United States.

⊥ Department of Internal Medicine, University of Texas Southwestern Medical Center, Dallas, Texas 75390, United States. Department of Microbiology, University of Texas Southwestern Medical Center, Dallas, Texas, 75390, United States.

*Corresponding author

Corresponding author:

Carolyn R. Bertozzi. Email: bertozzi@stanford.edu

Supplementary Figures, Schemes, and Tables

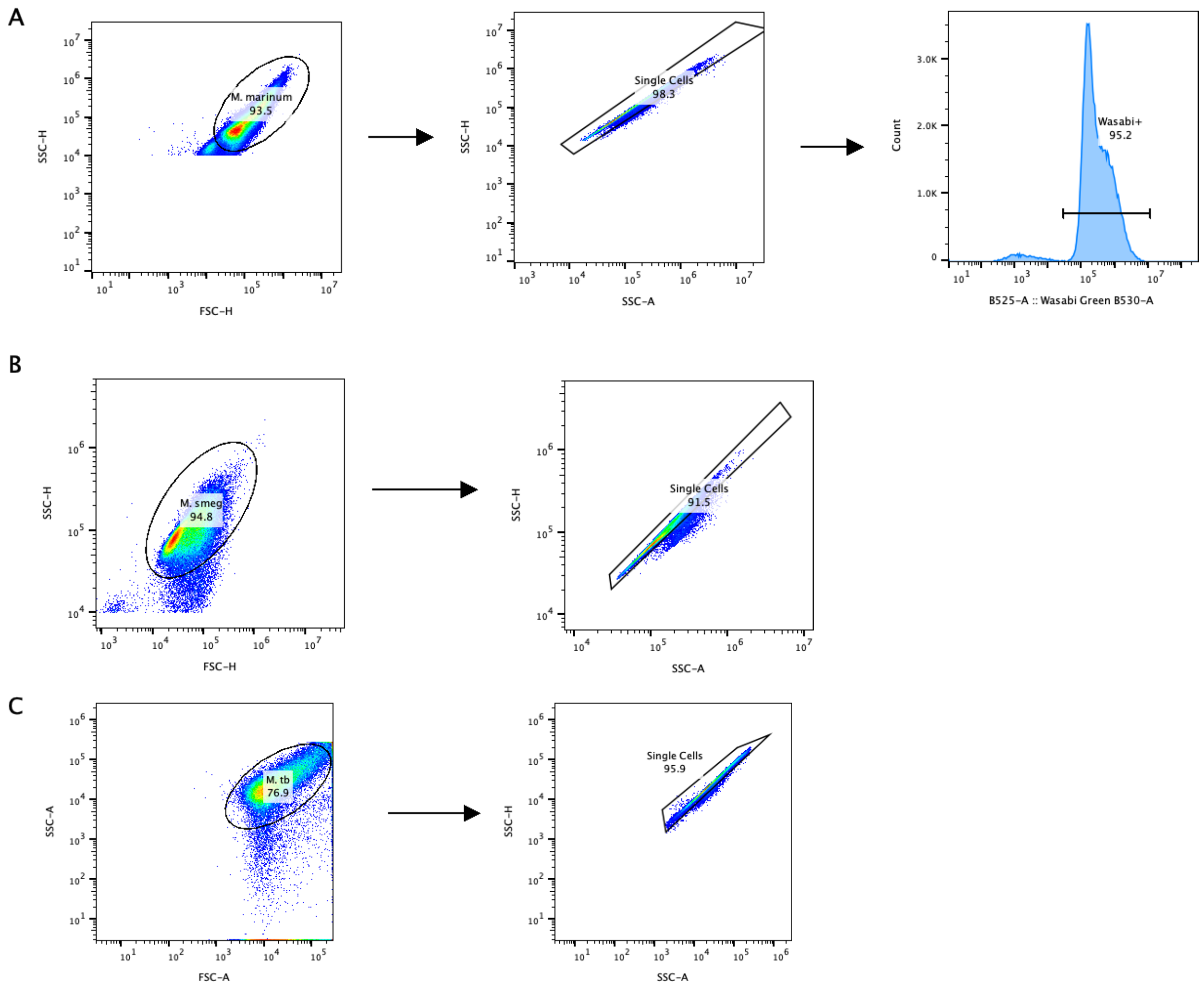

Figure S1. Gating strategy for flow cytometry experiments. (A) *M. marinum* expressing the blue fluorescent protein (wasabi) were selected from a side scatter-height vs. forward scatter-height plot. Single cells were selected in a side scatter-height vs. side scatter-area plot. The *M. marinum* expressing wasabi were then selected by gating on a histogram and statistics were then calculated using this population. (B) *M. smegmatis* were first selected from a side scatter-height vs. forward scatter-height plot. Single cells were selected in a side scatter-height vs. side scatter-area plot and statistics were calculated using this population. (C) *M. tuberculosis* cells were first selected from a side scatter-area vs. forward scatter-area plot. Single cells were selected in a side scatter-height vs. side scatter-area plot and statistics were calculated using this population.

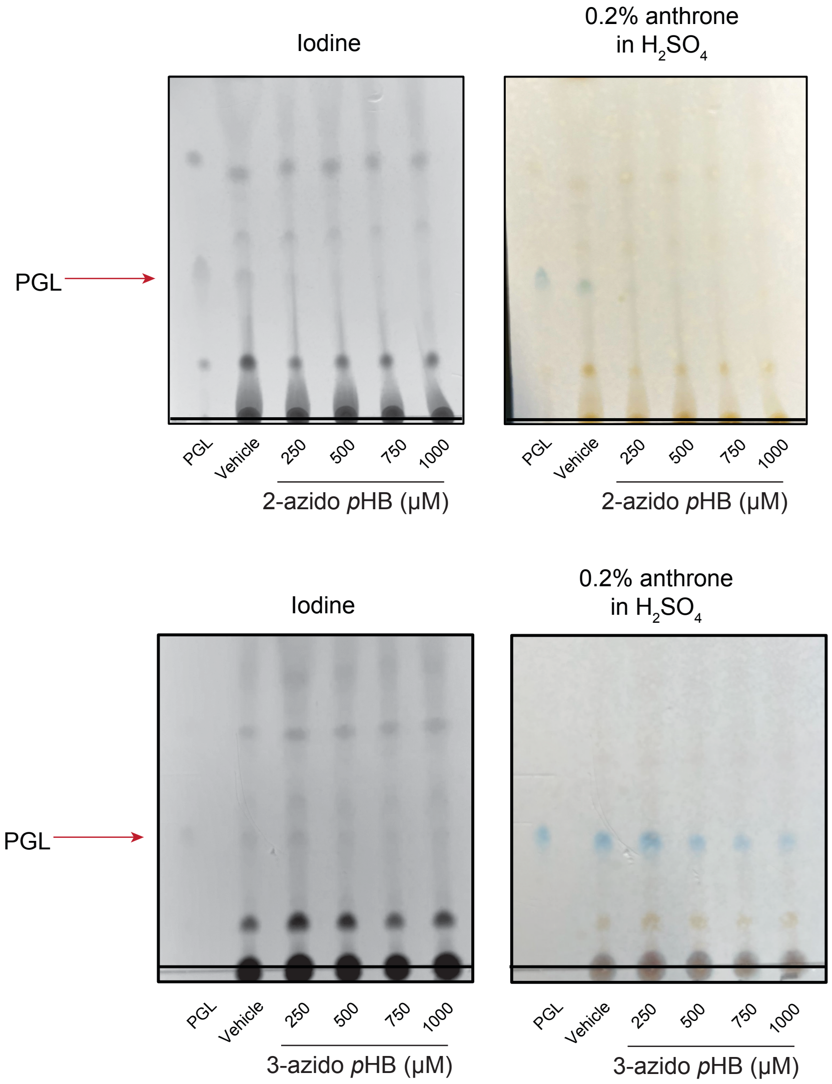

Figure S2. TLC plates of crude lipids extracted from *M. marinum* cultured with 2- or 3-azido *p*HB. 200 µg of crude lipids and 20 µg of purified PGL were loaded onto TLC plates and developed using 8:2 toluene:acetone. Plates were stained in 0.2% anthrone in H_2SO_4. The anthrone stain is used to identify compounds with sugars which will be revealed as a royal blue color. The iodine stain is a general stain used for total lipid identification. TLCs were imaged on a ChemiDoc MP imaging system using 590 nm wavelength.

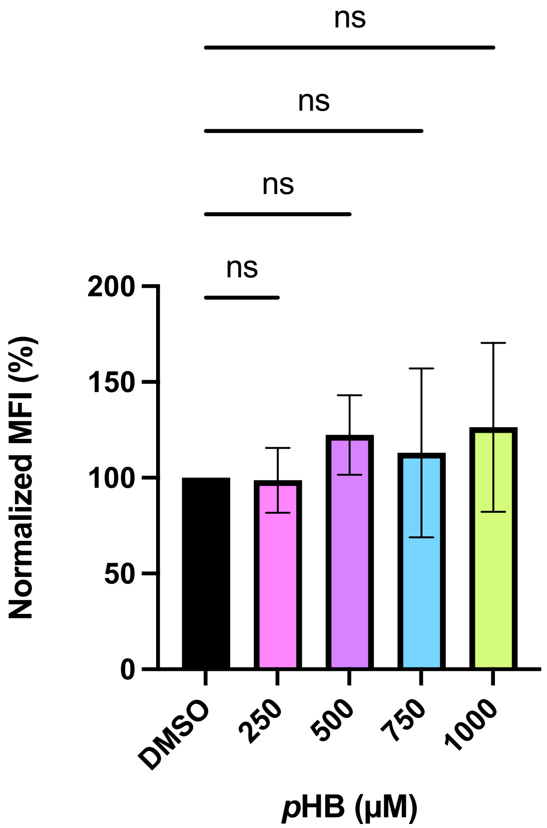

Figure S3. *M. marinum* cultured in various concentrations of *p*HB followed by treatment with DBCO-647 and analyzed by flow cytometry. General procedure for metabolic labeling was followed. Flow cytometry analysis represents three independent replicates. Relative MFI is normalized to DMSO control. Statistical analysis was performed using a one-way analysis of variance (ANOVA) followed by a Dunnett's multiple comparisons test. Significance is represented as follows: *$p \leq 0.05$, **$p < 0.01$, ***$p < 0.001$, ****$p < 0.0001$, and ns (not significant) for $p > 0.05$.

Supplementary Table 1

PGL				PGL-N3				
neutral molecule formula	[M+NH4]+ calculated m/z	[M+NH4]+ detected m/z	intensity (area)	neutral molecule formula	[M+NH4]+ calculated m/z	[M+NH4]+ detected m/z	mass error ppm	intensity (area)
C94H174O10	1481.3445	1481.344	1,038,938±110,812	C94H173N3O10	1522.3459	not detected		
C95H176O10	1495.3602	1495.359	712,617±48,059	C95H175N3O10	1536.3616	1536.365	2.3	66,001±645
C95H178O10	1497.3758	1497.375	304,183±19,881	C95H177N3O10	1538.3772	not detected		
C96H178O10	1509.3758	1509.376	1,548,098±124,234	C96H177N3O10	1550.3772	not detected		
C96H180O10	1511.3915	1511.390	337,981±11,557	C96H179N3O10	1552.3929	1552.403	6.8	49,841±5,499
C97H180O10	1523.3915	1523.393	4,417,171±278,616	C97H179N3O10	1564.3929	1564.394	0.6	206,331±3,679
C97H182O10	1525.4071	1525.407	753,652±63,171	C97H181N3O10	1566.4085	not detected		
C98H182O10	1537.4071	1537.407	1,719,998±124,695	C98H181N3O10	1578.4085	not detected		
C98H184O10	1539.4228	1539.424	2,776,249±166,514	C98H183N3O10	1580.4242	1580.424	0.1	160,966±8,394
C99H184O10	1551.4228	1551.421	4,578,012±364,026	C99H183N3O10	1592.4242	1592.435	6.8	36,801±6,232
C99H186O10	1553.4384	1553.436	952,323±71,916	C99H185N3O10	1594.4398	not detected		
C100H186O10	1565.4384	1565.437	1,123,848±71,813	C100H185N3O10	1606.4398	1606.449	5.8	20,635±2,192
C100H188O10	1567.4541	1567.456	4,640,701±309,886	C100H187N3O10	1608.4555	1608.452	2.2	29,151±5,547
C101H188O10	1579.4541	1579.455	3,088,382±163,416	C101H187N3O10	1620.4555	not detected		
C101H190O10	1581.4697	1581.464	315,240±16,810	C101H189N3O10	1622.4711	not detected		
C102H190O10	1593.4697	1593.469	496,021±55,504	C102H189N3O10	1634.4711	not detected		
C102H192O10	1595.4854	1595.486	1,509,159±100,619	C102H191N3O10	1636.4868	not detected		

Table S1. HPLC-MS positive mode analysis of PGL and PGL-N_3 from the extracted lipids of *M. marinum* grown in media supplemented with 750 µM 3-azido *p*HB. The known PGLs were detected as ammonium adducts, $[M + NH_4]^+$. For PGL-N_3, the theoretical molecular formula and mass were deduced by replacement of a proton for N_3. Seven PGL-N_3 species were identified by near coelution with PGL and matching the calculated masses to predict masses with errors less than 10 ppm.

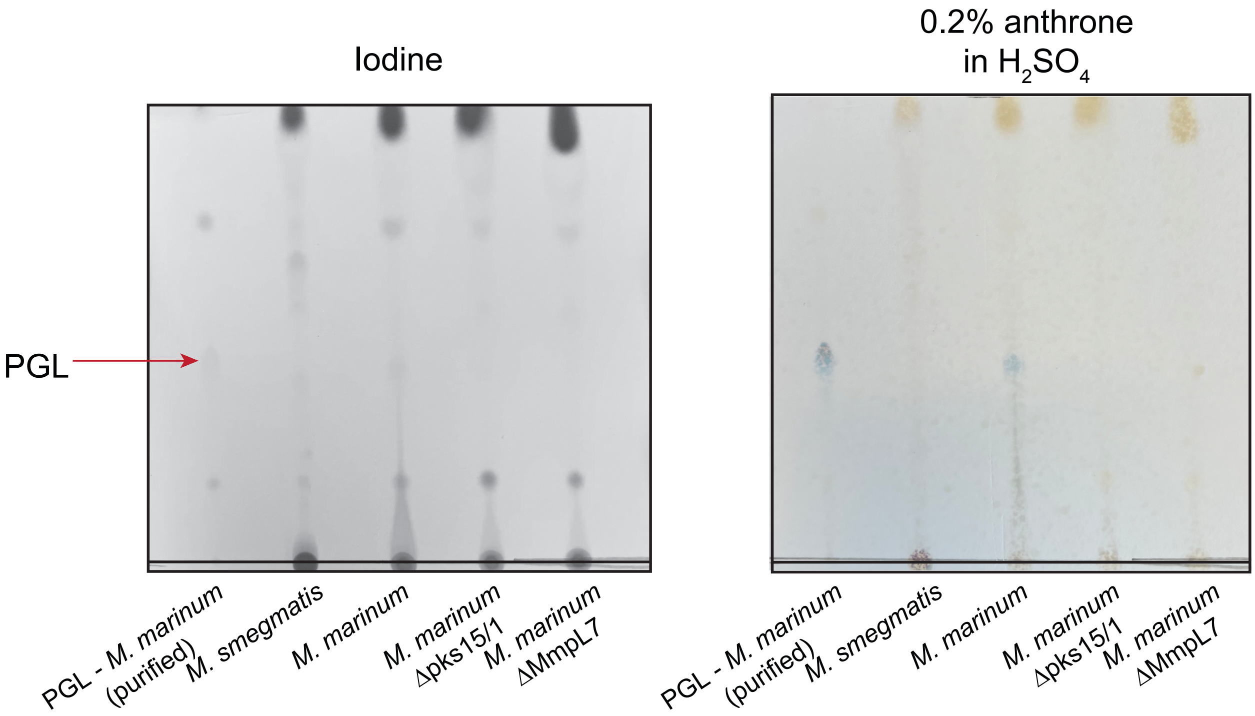

Figure S4. Crude lipid extracts from PGL-deficient *M. smegmatis*, PGL-producing WT *M. marinum*, and PGL-deficient *M. marinum* mutants indicate presence or absence of PGL. Purified PGL from M. marinum was used as a standard. Crude lipid extracts (100 µg) and purified PGL- *M. marinum* (5 µg) were loaded onto a silica gel 60 TLC plate and developed in 8:2 toluene:acetone. Compounds with sugars are visualized as a blue color in the anthrone stain. The iodine stain is a general stain used to visualize total lipids. TLCs were imaged on a ChemiDoc MP imaging system using 590 nm wavelength.

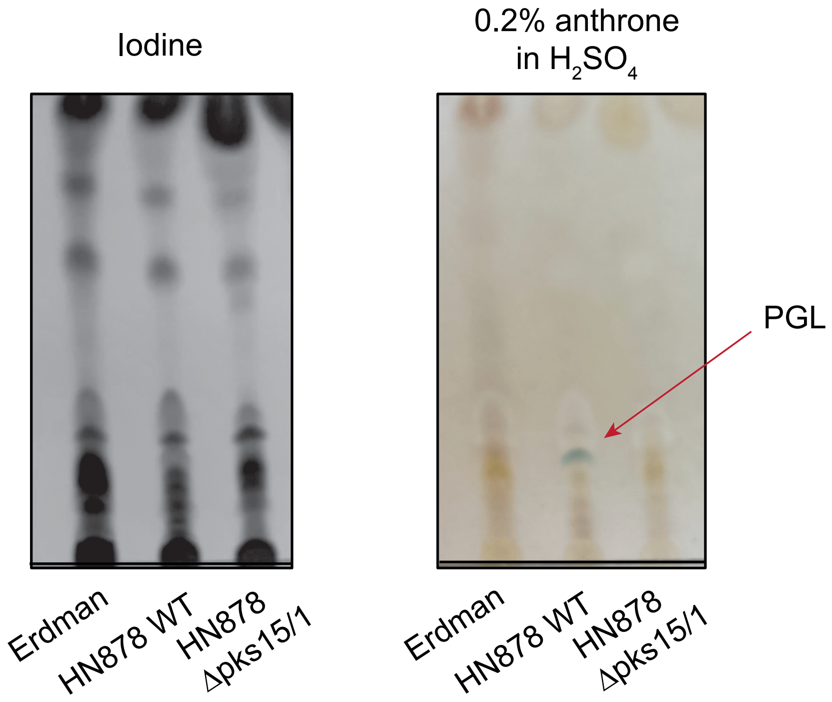

Figure S5. Crude lipid extracts from *M. tb* strains Erdman, HN878 WT, and HN878 Δpks15/1. Crude lipid extracts (100 µg) were loaded onto a silica gel 60 TLC plate and developed in 95:5 chloroform:methanol. Compounds with sugars are revealed as a blue color in the anthrone stain. The iodine stain is a general stain used to check for total lipids. TLCs were imaged on a ChemiDoc MP imaging system using 590 nm wavelength.

Supplementary methods

General methods for synthesis. Materials and reagents were obtained from commercial sources without further purification unless otherwise noted. Analytical TLC was performed on Millipore Sigma glass-backed Silica gel 60 F254 plates. Prep TLC was performed on Millipore Sigma glass-backed 2 mm thick Silica gel 60 F_{254} plates with concentration zone. TLC was analyzed using iodine adhered to silica and 0.2% anthrone in H_2SO_4 stain. Column chromatography was performed using normal-phase silica gel 60. ^{1}H and ^{13}C NMR spectra were obtained using a Brucker Neo-500 MHz instrument with chemical shifts in ppm (δ) referenced to solvent peaks. Flow cytometry analysis of *M. marinum* was performed on a Novocyte Penteon flow cytometer at the Stanford shared FACS facility. Flow cytometry analysis of *M. tuberculosis* BD FACSCalibur cytometer at UT Southwestern. High resolution mass spectrometry characterization of azide compounds was performed on an Thermo Orbitrap Fusion nano LC/MS instrument at the Stanford Mass Spectrometry facility. FRAP was performed at the Stanford microscopy facility on an inverted Zeiss 780 multiphoton laser scanning confocal microscope. Lipidomics analysis of crude extracts was performed on a reversed-phase Agilent 1260 series HPLC system and an Agilent 6546 Accurate-Mass Q-TOF mass spectrometer.

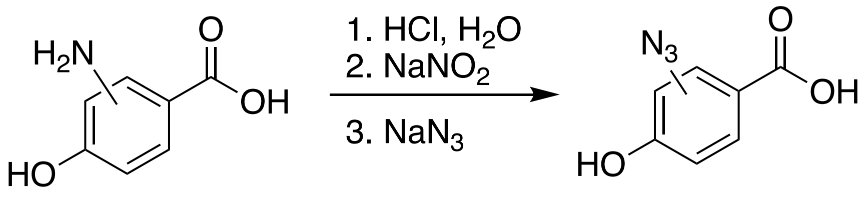

Scheme S1. Synthesis of 3- or 2- azido *p*HB.

General procedure for synthesis of 3- and 2-azido *p*HB. <u>*WARNING: the synthesis of azide compounds is dangerous and can result in explosions and/or production of toxic gases. It is recommended to synthesize these molecules at small scale (i.e. 1 mmol) behind a blast shield. Proceed with caution.*</u>

Aniline (2- or 3-amino *p*HB) starting materials (0.150 g, 1 mmol) were suspended in 2.5 mL of water in a round bottom flask. The round bottom flask was submerged in an ice bath and conc. HCl (0.25 mL) was added slowly. The flask was stirred for 10 minutes. Next, $NaNO_2$ (0.076 g, 1 mmol) was added to the flask and stirred for 10 minutes. Then NaN_3 (0.085 g, 1.3 mmol) was added to the flask and stirred for 30 minutes. After 30 minutes the flask was removed from the ice bath and warmed to room temperature. The reaction was monitored by normal-phase TLC using a 1:1 acetone:hexanes mobile phase. After ~2 hrs when the starting material was consumed, an additional 10 mL of

water was added to the flask and the product was extracted with 3 x 10 mL of ethyl acetate. The organic layer was then washed with brine (saturated NaCl solution) and dried using magnesium sulfate. The crude product was purified by silica gel chromatography using a gradient solvent system of acetone:hexanes. The product was achieved as an off-white/yellow solid. Yield of 2- azido *p*HB: 110 mg, 65%. Yield of 3-azido *p*HB: 0.131 g, 75%.

3-azido *p*HB: ^{1}H NMR (500 MHz, d_6-DMSO): δ 7.61 (dd, *J* = 2.2, 10 Hz, 1 H), 7.4 (d, *J* = 2.2 Hz, 1 H), 6.94 (d, *J* = 9.9, 1 H). ^{13}C NMR (500 MHz, d_6 DMSO): 166.52, 154.53, 127.82, 125.78, 122.32, 121.94, 116.07. HR ESI MS: calculated for $C_7H_5N_3O_3[-H]^-$ *m/z*, 178.0247; found 178.0255.

2-azido *p*HB: ^{1}H NMR (500 MHz, d_6-DMSO): δ 10.52 (s, 1H), 7.71 (d, J = 5, 1H), 6.64 (m, 2H). ^{13}C NMR (500 MHz, d_6-DMSO): δ 165.75, 161.77, 141.30, 133.88, 114.00, 112.44, 107.09. HR ESI MS: calculated for $C_7H_5N_3O_3[-H]^-$ *m/z*, 178.0258; found 178.0259.

NMR spectra

3-azido *p*HB: ^{1}H NMR (d^{6}-DMSO, 500 MHz)

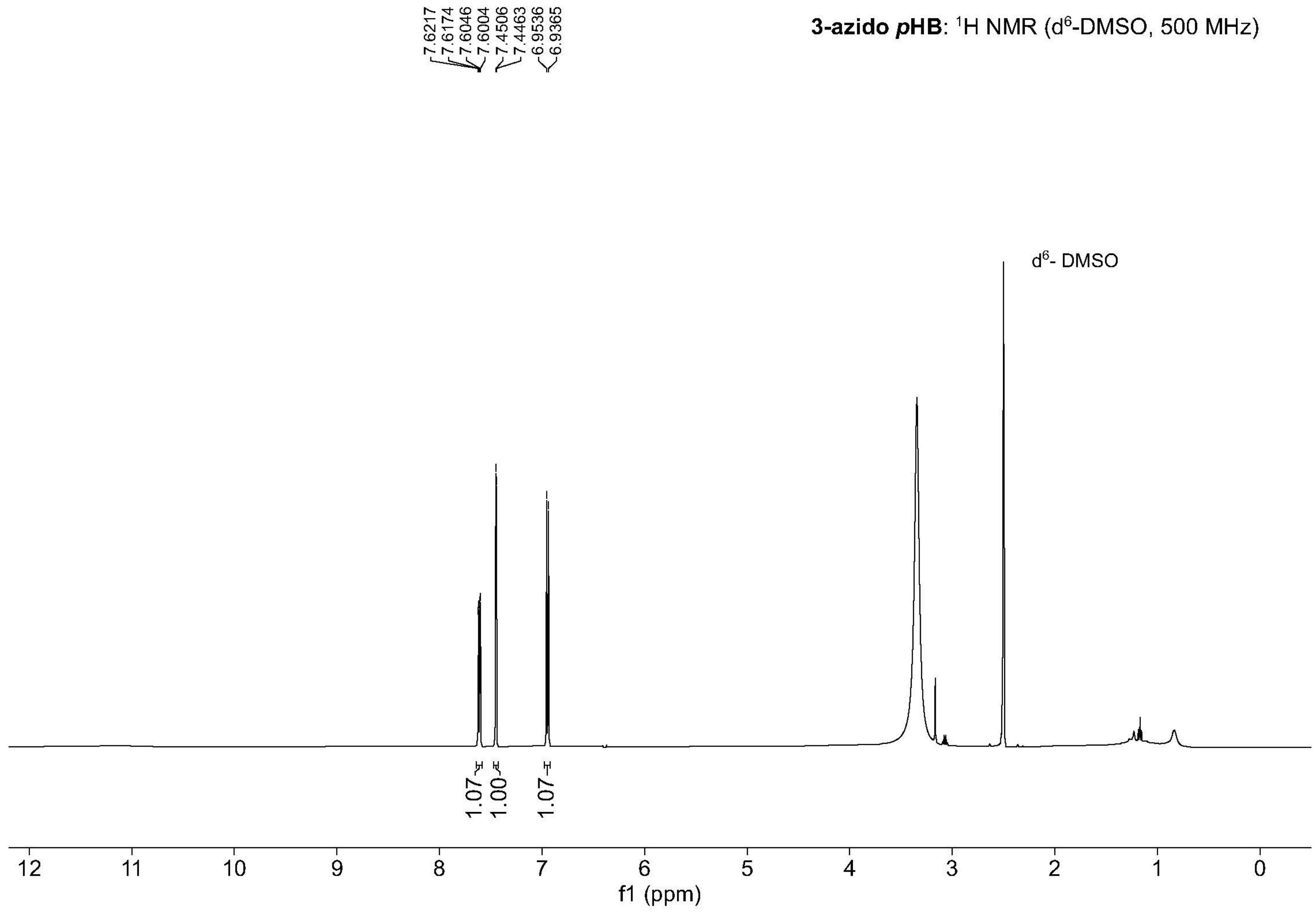

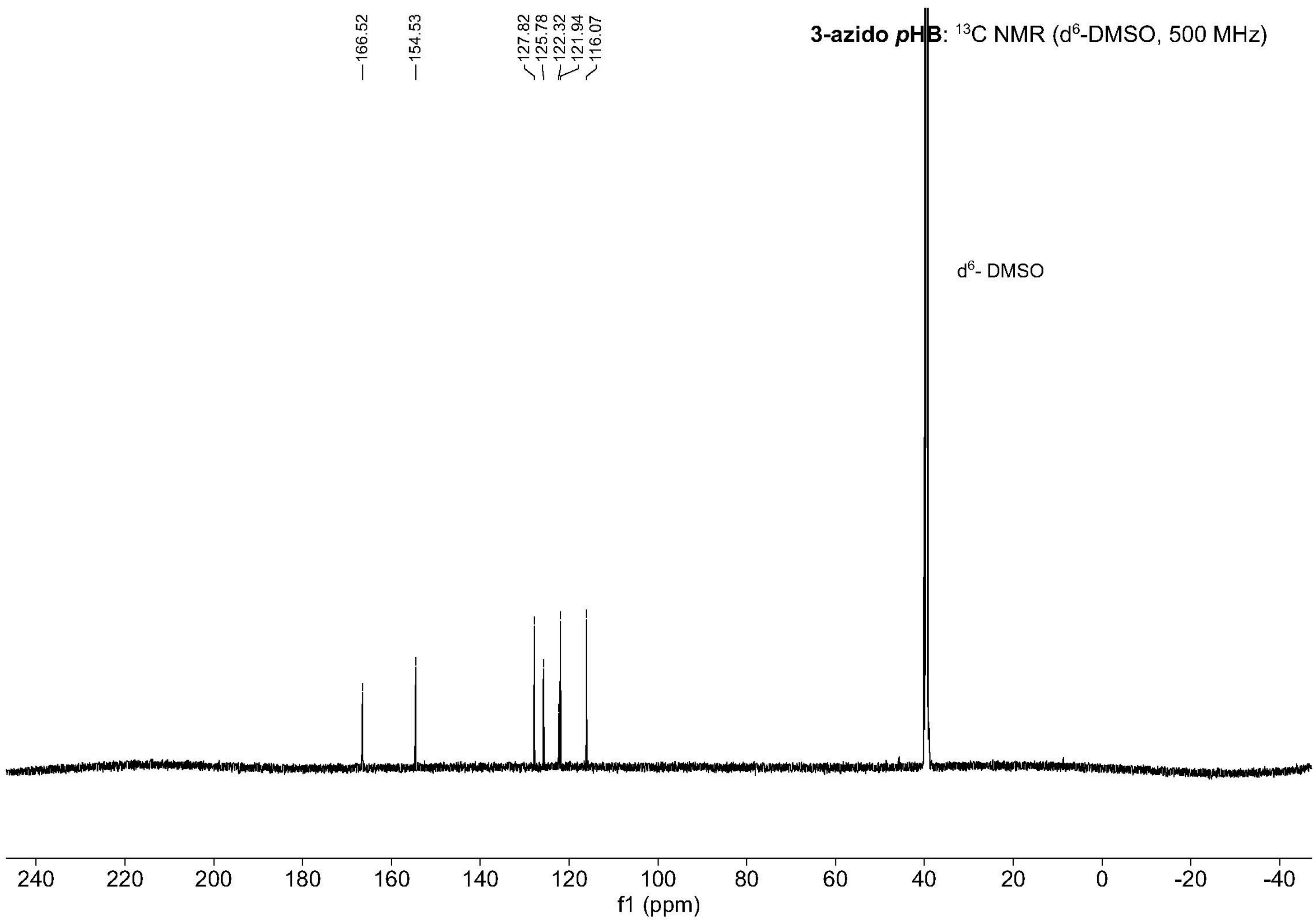

3-azido pHB: 13C NMR (d6-DMSO, 500 MHz)
166.52
154.53
127.82
125.78
122.32
121.94
116.07
d6- DMSO
240
220
200
180
160
140
120
100
80
60
40
20
0
-20
-40
f1 (ppm)

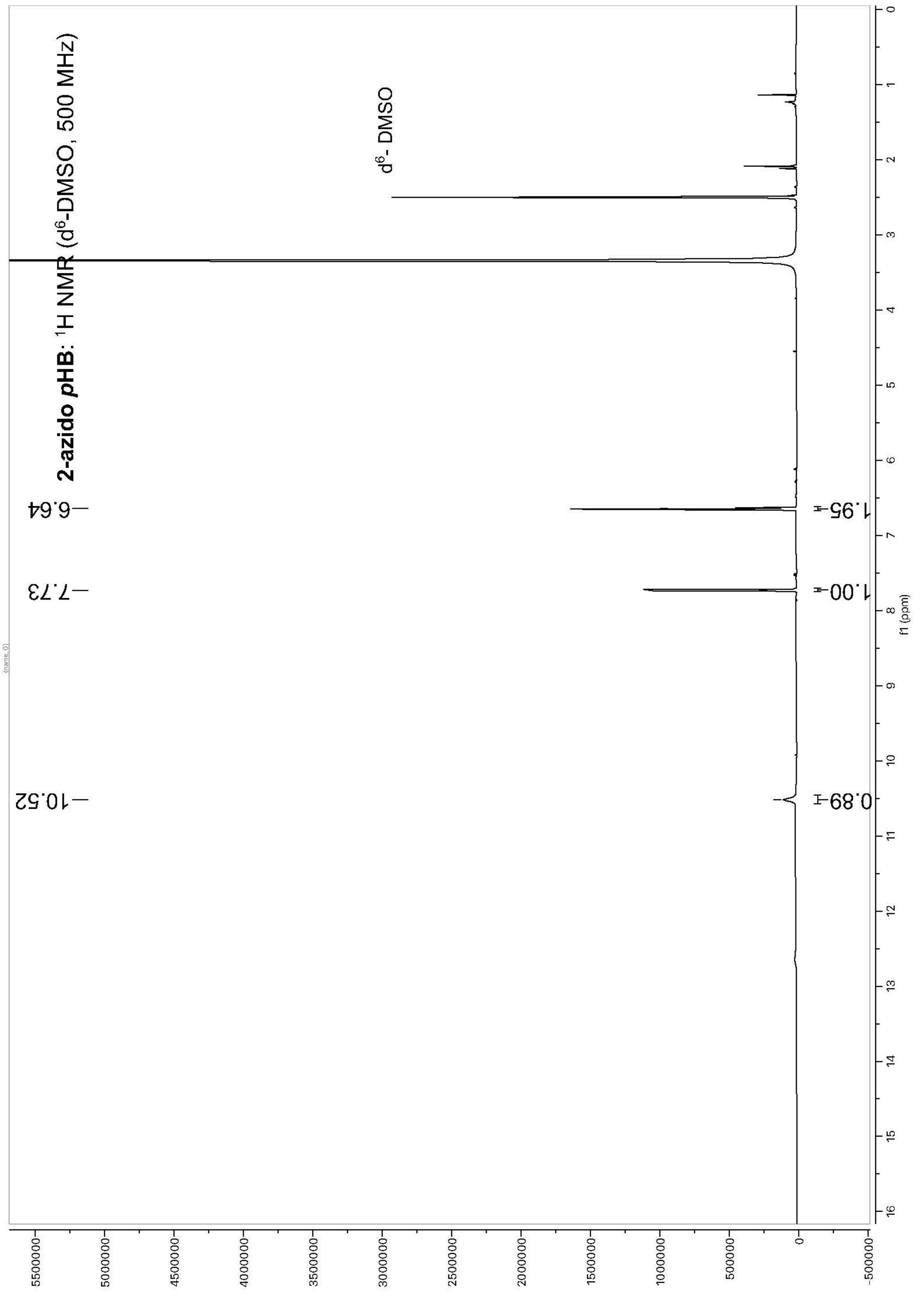

2-azido *p*HB: ^{1}H NMR (d^6-DMSO, 500 MHz)
d^6- DMSO
10.52
7.73
6.64
0.89
1.00
1.95
f1 (ppm)

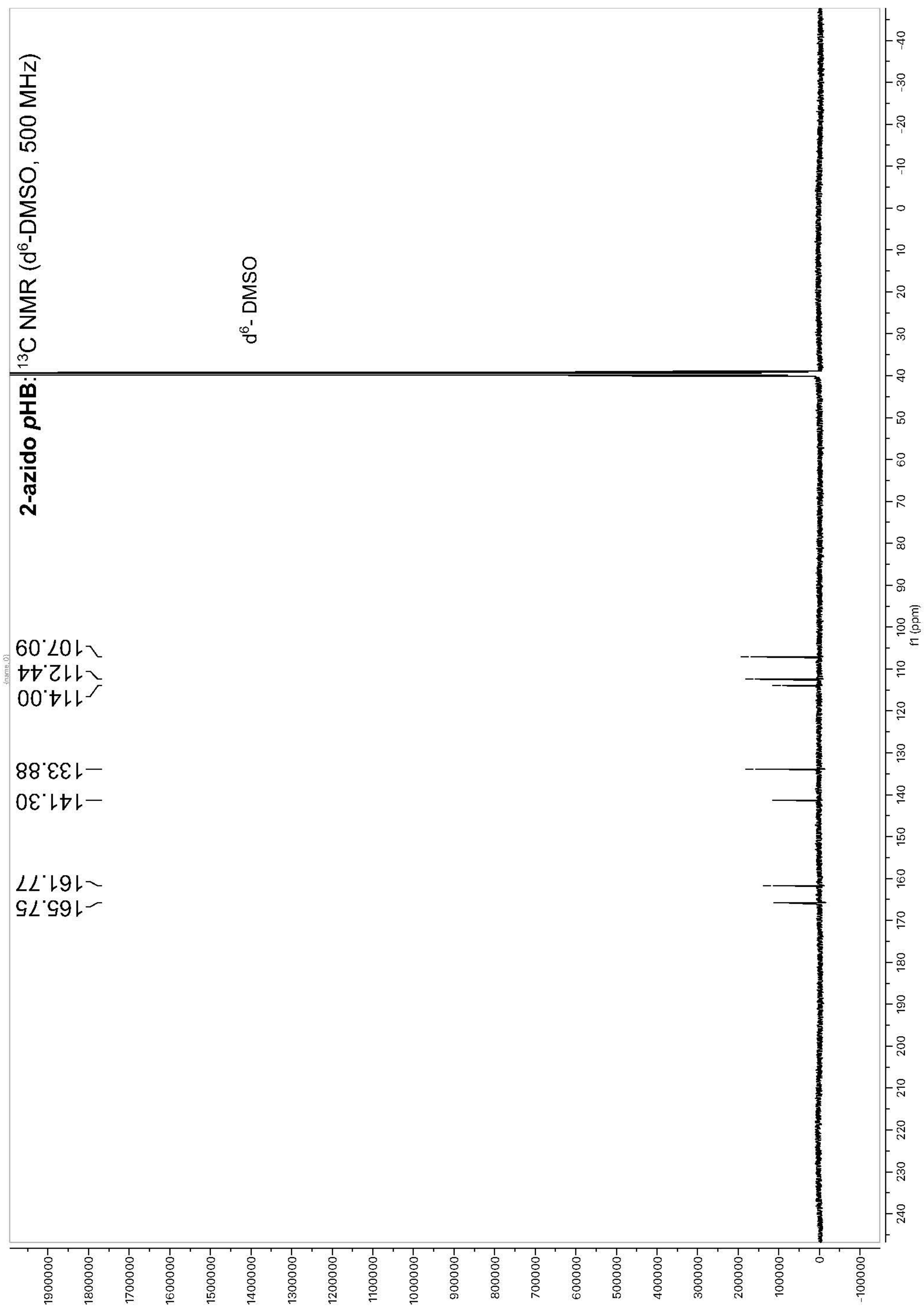
2-azido pHB: 13C NMR (d6-DMSO, 500 MHz)
d6- DMSO
107.09
112.44
114.00
133.88
141.30
161.77
165.75
f1 (ppm)
